# Use of the Electrostatic Classification Method to Size 0.1 μm SRM Particles—A Feasibility Study

**DOI:** 10.6028/jres.096.006

**Published:** 1991

**Authors:** Patrick D. Kinney, David Y.H. Pui, George W. Mulliolland, Nelson P. Bryner

**Affiliations:** University of Minnesota, Minneapolis, MN 55455; National Institute of Standards and Technology, Gaithersburg, MD 20899

**Keywords:** aerosol generator, atomizers, condensation nuclei counters, electrical mobility, particle size, polystyrene latex spheres

## Abstract

The use of the electrostatic classification method for sizing monodisperse 0.1 μm polystyrene latex (PSL) spheres has been investigated experimentally. The objective was to determine the feasibility of using electrostatic classification as a standard method of particle sizing in the development of a 0.1 μm particle diameter Standard Reference Material (SRM). The mean particle diameter was calculated from a measurement of the mean electrical mobility of the PSL spheres as an aerosol using an electrostatic classifier. The performance of the classifier was investigated by measuring its transfer function, conducting a sensitivity analysis to verify the governing theoretical relationships, measuring the repeatability of particle sizing, and sizing NIST SRM 1691, 0.269 μm and NIST SRM 1690, 0.895 μm particles. Investigations of the aerosol generator’s performance focused on the effect of impurities in the particle-suspending liquid on the resulting particle diameter.

The uncertainty in particle diameter determined by electrical mobility measurements is found to be −3.3% to +3.0%. The major sources of uncertainty include the flow measurement, the slip correction, and a dependence of particle size on the aerosol flow rate. It was found that the classifier could be calibrated to indicate the correct size to within 0.1% for both SRM particle sizes if the defined classification length is decreased by 1.9%.

## 1. Introduction

This study assesses the accuracy of electrostatic classification for measuring the diameter of 0.1 μm polystyrene latex (PSL) spheres produced as an aerosol by atomizing a suspension of PSL spheres in water. The PSL spheres used in this study were produced by emulsion polymerization by Dow Chemical Company^1^ and the nominal size as measured at Dow by transmission electron microscopy is 0.109 μm. This study is motivated by the need to develop an accurate 0.1 μm particle size standard. This size standard is important for improving particle sizing accuracy by electron microscopy, light scattering, and by other methods. A particle diameter of 0.1 μm is in the size range of combustion generated particles, contamination particles of concern in the semiconductor industry, air pollutant particulates, viruses, and various manufactured particulates such as carbon black and fumed silica.

The electrostatic classifier is a widely used instrument in aerosol research for both particle sizing and for generation of monodisperse aerosols over the size range 0.005 to 1.0 μm. The basic physical principle of the classifier is that the velocity of a charged spherical particle in an electric field is directly related to the diameter of the particle. A charged aerosol enters near the outer circumference of the classifier and particles with a narrow range in electrical mobility exit through a slit in the center electrode. The mobility distribution is determined by measuring the number concentration exiting the slit as a function of the electrode voltage.

The theory of the classifier operation and its performance have been extensively studied [1–7]. In regard to sizing PSL spheres with the classifier, Kousaka et al. [8], compared measurements of PSL spheres made with the electrostatic classifier to measurements made with a sedimentation method, and a balance method using a Millikan type cell. The three measurements were found to agree within a few percent for 0.2 to 1 μm particles, but measurements were not conducted for particles smaller than 0.2 μm in diameter.

The determination of the accuracy of a measurement method requires that all the physical variables entering into the particle size equation be accurately known. The two key physical variables for the electrostatic classifier are the volumetric flow rate and the electrode voltage. The procedure used at NIST for these two calibrations is described in section 3.2.1. An important element in assessing the accuracy of an instrument is the verification that the instrument behaves according to the governing equation. The verification procedure outlined in section 3.2.3 included comparison with the theory [1] and the use of two classifiers in tandem [9]. Further verification of the classifier performance is contained in section 3.2.4, where the measured and predicted sensitivity of the classifier peak voltage to a change in the flow volume and the operating pressure are compared. Another way the accuracy of the classifier was established was by measuring two primary calibration standards for particle size: 0.269 μm (NIST SRM 1691) and 0.895 μm (NIST SRM 1690). The results of this comparison are contained in section 3.2.6.

The method for generating the PSL sphere aerosol involved atomizing a suspension of PSL spheres dispersed in water. The non-volatile impurities in PSL sphere suspension result in a residue thickness on the PSL sphere. Significant effort was involved in minimizing the droplet size produced by the atomizer system, section 3.3.1, and in quantifying the amount of impurity in the dilution water, section 3.3.2, and in the original, undiluted suspension, section 3.3.3.

In section 4, the Discussion section, a comparison is made between the results of this study and two other studies [10,11] that focused on the accurate measurement of the same batch of Dow 0.109 μm PSL spheres.

## 2. Experimental Apparatus

**Figure 1:**
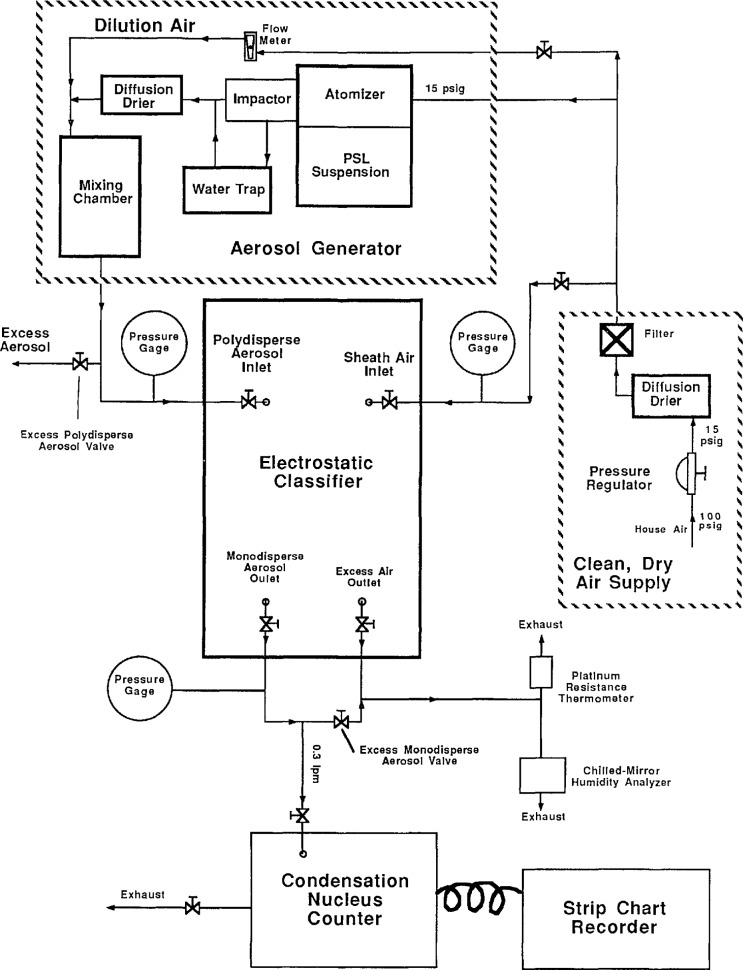
Apparatus for particle sizing with the electrostatic classifier.

Figure 1. shows a schematic diagram of the instrumentation used in this study. The major components are the atomizer, the electrostatic classifier, and the condensation nucleus counter. A PSL aerosol is produced by atomizing a suspension of PSL spheres in water. After conditioning, the particles are passed through the electrostatic classifier. By monitoring the number concentration with the nucleus counter versus the mobility setting of the classifier, the mean electrical mobility of the particles is determined. The mean size is then determined from the particle size dependence of the electrical mobility. A more detailed description of each instrument follows.

### 2.1 Aerosol Generation

**Figure 2:**
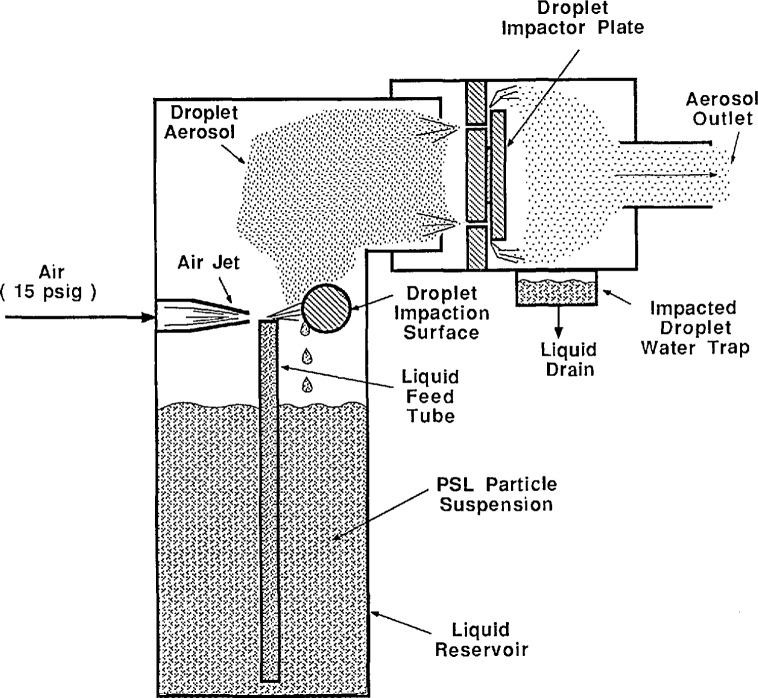
Atomizer with droplet impactor.

The PSL spheres are aerosolized with an atomizer, shown in figure 2, consisting of a 15 psig air jet impinging on the end of a liquid feed tube. The opposite end of the feed tube is submerged in a suspension of PSL spheres in water. The vacuum produced by the air jet draws the particle suspension through the capillary tube and into the air jet. The jet atomizes the PSL particle suspension producing an aerosol of droplets. Some of the droplets produced contain PSL spheres while other droplets are “empty.” The droplets evaporate as they flow through a diffusion drier and are diluted with clean, dry air. Droplets containing PSL spheres evaporate to form a PSL sphere with a slight surface residue. Droplets which do not contain a PSL sphere evaporate and form a small residue particle consisting of nonvolatile impurities present in the original particle suspension liquid. Thus, the resulting aerosol consists of potentially dirty PSL spheres and small impurity particles.

When a droplet containing a PSL sphere evaporates, any non-volatile impurities in the liquid remain to form a thin layer of residue on the particle surface. The residue formed on the surface has a finite thickness and produces a systematic error in the measurement of particle diameter. To reduce the concentration of impurities in the particle suspension, de-ionized/filtered water was used to suspend the PSL spheres. The larger droplets evaporate leaving a larger impurity residue on the particle surface. To minimize this effect, an impactor with a cut point of about 0.5 μm was placed at the outlet of the atomizer. The effect of impurity concentration on the size of 0.1 μm PSL spheres has been experimentally investigated in this study.

### 2.2 The Electrostatic Classifier

**Figure 3:**
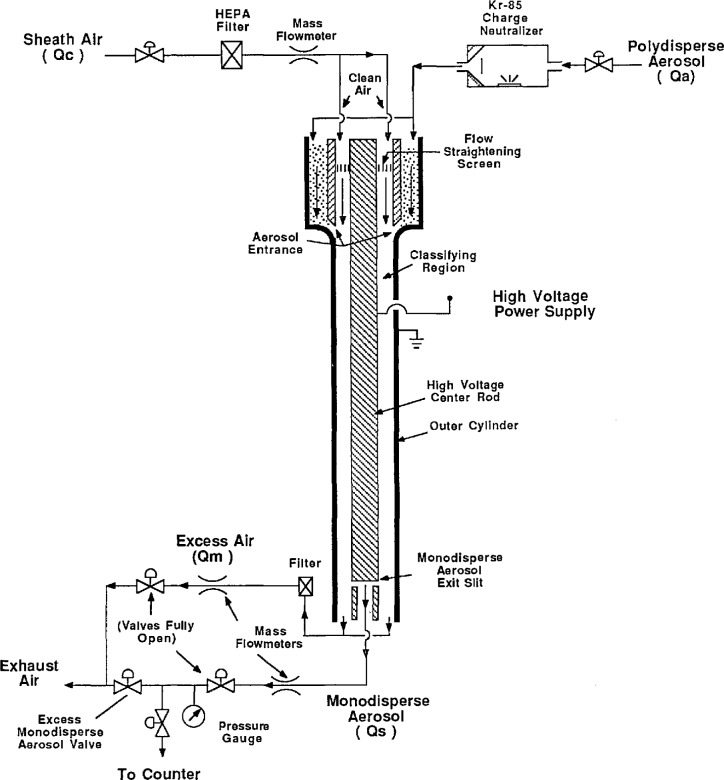
Electrostatic classifier.

The electrostatic classifier used in this project is a commercially available instrument (TSI, Inc., Model 3071). Figure 3 shows a schematic diagram of the instrument. The classifying region is bounded by a stainless steel outer cylinder with an inner diameter of 3.916 cm, and a coaxial, stainless steel center rod with an diameter of 1.874 cm. The center rod is connected to a variable (0 to −11,000 V) negative dc power supply, and the outer cylinder is grounded. By varying the center rod voltage, the electric field in the annular region can be varied from 0 to about 11,000 V/cm.

Clean sheath air, after passing through a fine-mesh flow straightening screen at the top of the classifier, flows axially through the annular region along the center rod. A smaller, polydisperse aerosol flow enters through an axisymmetric opening along the outer cylinder. The clean air flow forces the aerosol to flow downward in a thin layer on the outer wall of the classifying region. It is essential that these two streams merge smoothly without mixing. Near the bottom of the classifying region, a slit on the center rod extracts a fraction of the air flow consisting of near-monodisperse (single sized) aerosol particles. The remainder of the air flow exits through the end of the annular region as excess air. The length of the classifying region (44.44 cm) is defined as the axial distance from the aerosol entrance to the aerosol exit at the slit in the center rod.

Before entering the classifying region, the particles are sent through a charge neutralizer. The neutralization occurs through interaction with bi-polar gaseous ions (positive and negative ions) produced by radioactive Kr-85. The ions impart a bi-polar charge distribution on the aerosol particles. For particles with diameters around 0.1 μm, about 24% of the particles carry a single positive elementary charge and about 4% carry a double positive charge [12].

When the particles enter the classifying region, they are carried axially down the classifying region with the sheath air flow, and the particles carrying a positive charge move radially towards the center rod under the influence of the electric field. Negatively charged particles deposit on the inner surface of the outer cylinder. Within the classifying region, a particle rapidly reaches a steady radial velocity through equilibrium between the electric field force, and the opposing Stokes drag force. The radial velocity of the particle in the electric field is determined by the particle’s electrical mobihty, defined as the velocity a particle attains under the influence of a unit electric field.

The electrical mobility, *Z*_p_, of a singly-charged particle can be derived by equating the electric field force, *F*_ϵ_, with the Stokes drag force, *F*_d_:

$$\begin{array}{ll} \text{Stokes}\;\text{Drag}\;\text{Force}: & F_{d}=\frac{3\pi \mu VD_{p}}{C\left(D_{p}\right)}\\ \text{Electric}\;\text{Field}\;\text{Force}: & F_{\epsilon }=\epsilon E\\ \text{Electric}\;\text{Mobility}: & Z_{p}=\frac{V}{E}=\frac{\epsilon C\left(D_{p}\right)}{3\pi \mu D_{p}} \end{array}$$ (1)

Where

*V* = radial component of particle velocity
*E* = electric field strength
ϵ = elementary unit of charge
*C*(*D*_p_) = slip correction
μ = air viscosity
*D*_p_ = particle diameter.

As seen from eq (1), small particles have high electrical mobilities, and thus move with high radial velocities toward the center rod and deposit on its surface. Larger particles, with lower electric mobilities, are swept further down the classifying region before depositing on the center rod. Still larger particles are swept out the bottom of the classifier with the excess air. The monodisperse output of the classifier is extracted through a small slit on the center rod shown in figure 3. Only particles with electric mobilities within a narrow range have trajectories which bring them to the entrance of the slit. Particles reaching the entrance of the slit are removed from the classifying region by the air flow entering the slit. In this way the classifier extracts a narrow size range of particles from the broader size range of particles entering the classifying region.

#### 2.2.1 The Transfer Function

Knutson and Whitby [1] developed a theory for the classifier based on integrating the particle trajectory equations. The major result of their theory is an equation for the transfer funcfion, Ω, which is defined as the probability an aerosol particle that enters the analyzer will leave via the sampling flow given that the particle has a mobility *Z*_p_. A brief summary of their analysis is presented below.

Let *r* and *z* denote the radial and axial coordinates, respectively, within the mobility analyzer with *z* increasing in the direction of the main airflow. Let *u_r_*(*r*,*z*) and *u_z_*(*r*,*z*) be the radial and axial components of the airflow velocity. Similarly, let *E_r_*(*r,z*) and *E_z_*(*r*,*z*) be the components of the electric field. Neglecting particle inertia and Brownian motion, one obtains the following two first order differential equations for the particle path:

$$dr/dt=u_{r}+Z_{p}E_{r},$$ (2)

$$dz/dt=u_{z}+Z_{p}E_{z}.$$ (3)

To demonstrate the conjugate nature of the flow field and the electric field, Knutson and Whitby transformed to new coordinates, ψ, the stream function, and ϕ, the electric flux function.

$$\psi \left(r,z\right)=\int^{r,z}\left[ru_{r}dz-ru_{z}dr\right],$$ (4)

$$\phi \left(r,z\right)=\int^{r,z}\left[rE_{r}dz-rE_{z}dr\right].$$ (5)

They then demonstrate that the total differential of ψ+*Z*_p_ϕ is zero leading to the following significant result:

$$\psi =-Z_{p}\phi +\text{constant}.$$ (6)

Quoting Knutson and Whitby [1] “The particle moves in such a way that the ratio of the number of streamlines crossed to the number of electric field lines crossed is always equal to the particle electric mobility, *Z*_p_.”

**Figure 4:**
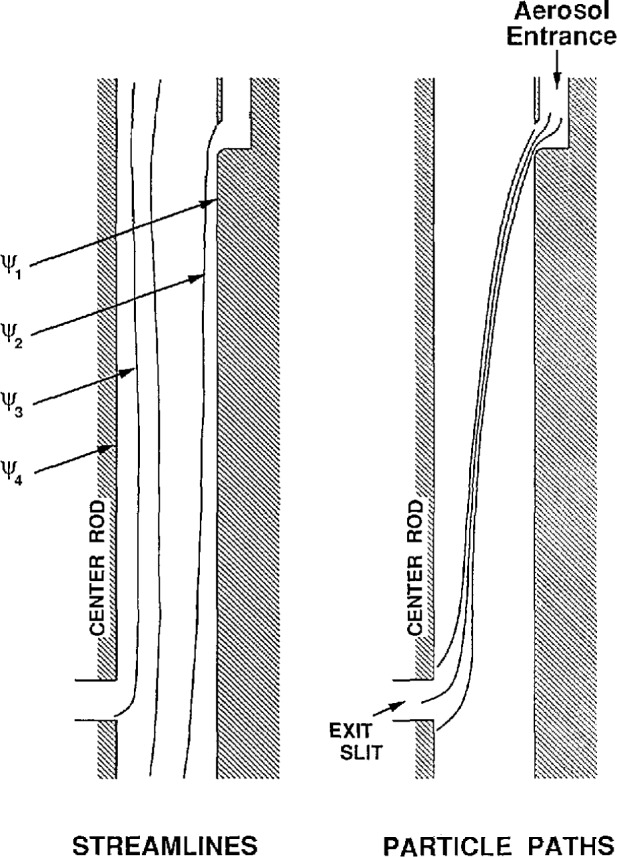
Schematic representation of mobility analyzer streamlines and particle paths.

The advantage of this method of analysis is that the stream function is closely related to the volumetric flow rate, which is an experimentally controlled variable. Representative streamlines are indicated in figure 4 and the corresponding flow variable is indicated below:

2π(ψ_2_−ψ_1_) = aerosol inlet volume flow rate, *Q*_a_
2π(ψ_4_−ψ_2_) = inlet sheath air volume flow rate, *Q*_c_
2π(ψ_4_−ψ_3_) = monodisperse aerosol volumetric flow rate, *Q*_s_
2π(ψ_3_−ψ_1_) = outlet excess air volume flow rate, *Q*_m_.

With the initial condition ψ = ψ_in_ at ϕ = ϕ_in_, eq (6) for the particle path becomes:

$$\psi =\psi_{\text{in}}-Z_{p}\left(\phi -\phi_{\text{in}}\right).$$ (7)

At ϕ = ϕ_out_, ψ has the value ψ^*^, given by:

$$\psi^{*}=\psi_{\text{in}}-Z_{p}\Delta \phi ,$$ (8)

where Δϕ = ϕ_out_ − ϕ_in_. The electric field is vanishingly small in the aerosol entrance and at the exit slip so that ϕ is a constant ϕ_in_ throughout the entrance and ϕ_out_ throughout the exit slit.

The transfer function, Ω, is the probability that the particle will leave via the sampling slit, which can happen only if

$$\psi_{3}<\psi^{*}<\psi_{4}.$$ (9)

**Figure 5:**
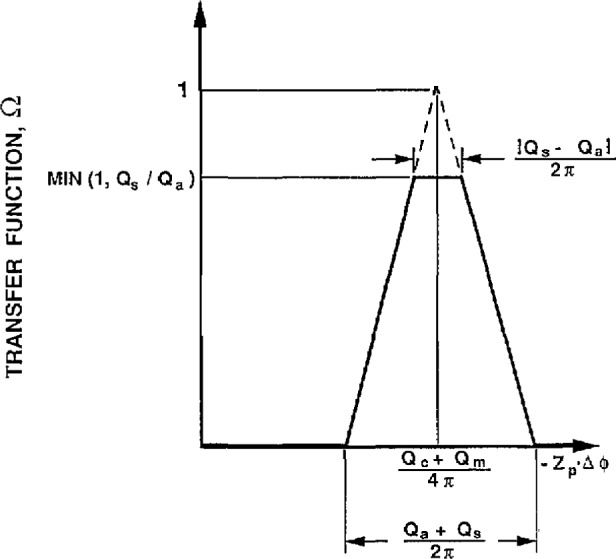
The mobility analyzer transfer function. The dashed curve corresponds to *Q*_s_ = *Q*_a_.

The probability, Ω, is therefore equal to the fraction of the interval (ψ_1_−*Z*_p_Δϕ, ψ_2_−*Z*_p_Δϕ) which is intercepted by the interval (ψ_3_,ψ_4_). The results of carrying out such an analysis, which is tedious but straightforward, are presented in figure 5.

There are several important features of the transfer function apparent from figure 5. If the aerosol inlet flow and the monodisperse sampling flow are equal, *Q*_a_ *= Q*_s_, the transfer function has a triangular shape with a sharp peak corresponding to a probability ft of 1. This is the best condition for obtaining accurate particle size.

For unequal flow rate, the transfer function has a trapezoidal shape. The origin of the top of the trapezoid can be understood intuitively from the following example. Suppose the inlet aerosol flow is less than the monodisperse sampling flow. Then there will be a range of voltages for the center rod for which all the inlet aerosol with mobility *Z*_p_ will be sampled by the monodisperse outlet. This implies that the transfer function is unity for a range of voltages thus leading to a flat top rather than a triangular shaped peak.

The actual measurements of mobility are made versus the voltage setting of the center rod. The relationship between Δϕ and the voltage *V* can be obtained using eq (5) and the expression for the radial and axial components of the electric field:

$$E_{z}=0,\;E_{r}=V/\left[r\ln\left(r_{2}/r_{1}\right)\right].$$ (10)

Performing the integration yield the following result:

$$\Delta \phi =VL/\ln\left(r_{2}/r_{1}\right).$$ (11)

The three features of figure 5 of greatest importance to the measurement of particle size are the centroid of the transfer function, and the upper and lower widths of the transfer function. Expressing the results in terms of the mobility, *Z*_p_, Knutson and Whitby [1] obtained the following expression for the centroid of the mobility band.

$$Z_{p}=\frac{Q_{c}+Q_{m}}{4\pi VL}\ln\left(r_{2}/r_{1}\right).$$ (12)

The range of the electrical mobility, Δ*Z*_p_, of particles exiting through the slit is given as:

$$\Delta Z_{p}=\frac{\left(Q_{a}+Q_{s}\right)}{2\pi VL}\ln\left(r_{2}/r_{1}\right).$$ (13)

The range in Δ*Z*_p_ corresponding to the upper portion of the trapezold in figure 5, which we denote as Δ*Z*_p_^*^ is given by

$$\Delta {Z_{p}}^{*}=\frac{\left|Q_{s}+Q_{a}\right|}{2\pi VL}\ln\left(r_{2}/r_{1}\right).$$ (14)

where

*Z*_p_ = particle electrical mobility
Δ*Z*_p_ = electrical mobility width at base
Δ*Z*_p_^*^ = electrical mobilitywidth of plateau region
*V* = voltage on the center rod
*L* = length from aerosol inlet to exit slit
*r*_2_ = radius of the outer cylinder (inside surface)
*r*_1_ = radius of the center rod.

Several assumptions were made in the development of these equations. The flow field is assumed to be laminar, axisymmetric, and incompressible; the electric field is assumed uniform, neglecting field distortions at the aerosol entrance and the sampling exit slit; particle inertia and Brownian motion are neglected; and the influence of space and image charges are assumed negligible.

#### 2.2.2 Measurement of the Electrical Mobility Distribution

By varying the voltage on the inner rod of the classifier, and measuring the concentration of the aerosol exiting through the monodisperse aerosol outlet, the distribution of the inlet aerosol’s electrical mobility can be measured. The resolution of this measurement can be controlled, as seen in eqs (12) and (13), by decreasing the ratio of aerosol flow rate to sheath flow rate. Using eq (1), the electrical mobility distribution can be converted to the size distribution of the inlet aerosol.

The classifier is instrumented with an adjustable voltage power supply and three mass flowmeters which control the sheath air, excess air, and monodisperse aerosol flow rates. The flowmeters operate by measuring the current needed to maintain a constant-temperature hot-wire element in the air flow and are sensitive to the mass of air passing the sensing element.

The calibration of a mass-sensing flowmeter can take the form of either an actual mass flow rate curve ([grams of air]/second vs meter voltage) or a volumetric flow rate curve ([liters of air]/second at *T,P* vs meter voltage) where *T* and *F* are the air temperature and pressure during calibration. As seen in eqs (12) and (13), measurements made with the classifier depend on volumetric flow rates. Since the classifier measures the flow rates using mass-sensing flowmeters, a correction must be made if the temperature and pressure of the air in the classifier differ from the temperature and pressure of the air used for the flowmeter calibration.

If the flowmeters are calibrated using dry air, the correction to the calibration for dry air can be obtained from the ideal gas equation, and is as follows:

$$Q_{\text{vol}}=Q_{\text{cal}}\cdot \frac{T_{\text{actual}}}{T_{\text{cal}}}\cdot \frac{P_{\text{cal}}}{P_{\text{actual}}}$$ (15)

where

*Q*_vol_ = volumetric flow rate
*Q*_cal_ = calibrated volumetric flow rate at *T*_cal_,*P*_cal_
*T*_cal_ = calibration temperature
*P*_cal_ = calibration pressure
*T*_actual_ = actual temperature inside classifier
*P*_actual_ = actual pressure inside classifier.

The correction to the flowmeter calibration for wet air is slightly different, and is described in section 3.2.2.

During operation of the classifier, the pressure, temperature and relative humidity of the air inside the classifier were measured, and the volumetric flow rate was calculated using eq (15). The temperature and relative humidity of the air inside the classifier were found by measuring the conditions of the air passing through the excess air line. The temperature was measured using a platinum resistance thermometer, and the relative humidity was measured using a chilled-mirror humidity analyzer.

The pressure inside the classifier was about 3.5×10^3^ Pa (36 cm H_2_O) above ambient for 333 cm^3^/s (20 L/min) sheath air flow. To minimize the gage pressure in the classifier, the excess air and monodisperse aerosol valves were left fully open, and flows were adjusted with the excess monodisperse aerosol valve (see fig. 3). The elevated pressure inside the classifier is required to exhaust the sheath flow through the flow straightening header at the bottom of the classifying region. The pressure inside the classifier is monitored by measuring the pressure in the monodisperse aerosol outlet line, and applying a slight correction, 150 Pa (1.5 cm H_2_O) for a 33.3 cm^3^/s (2 L/min) aerosol flow, to account for the pressure drop from the interior of the classifier to the pressure tap on the monodisperse outlet.

### 2.3 The Condensation Nucleus Counter

**Figure 6:**
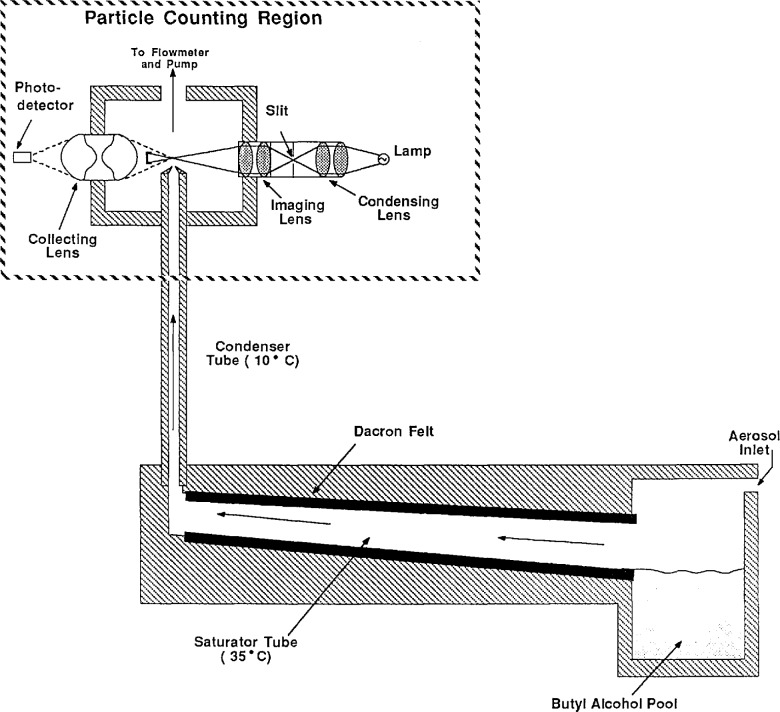
Condensation nucleus counter.

Figure 6 shows a schematic diagram of the condensation nucleus counter (CNC) (TSI, Inc., Model 3020). The instrument samples aerosol at a flow rate of 5 cm^3^/s and indicates the number concentration of the aerosol. The counting efficiency is nearly 100% for particles from about 0.02 to at least 0.1 μm [13].

The aerosol entering the counter passes through a chamber containing nearly saturated butyl alcohol vapor. The aerosol-alcohol vapor mixture is then passed through a cooled condensing tube causing the alcohol vapor to condense onto the particles. The condensing alcohol causes the particles to grow to a size easily detected with an optical counter at the exit of the condensing tube. In the optical particle counter, the particles pass through a focused light beam and scatter light onto a photodetector. In the single particle counting mode, used for lower particle concentrations, counting of individual pulses from the photodetector provides particle concentration. In the concentration mode, used for high particle concentrations, the analog level of the photodetector is calibrated to provide particle concentration. In general, since the single particle counting mode does not require calibration, its concentration measurements are considered more accurate. For sizing PSL spheres, the concentration was kept low enough to use the single particle counting mode. The PSL particle concentrations downstream of the classifier were maintained by adjusting the concentration of the PSL suspension used in the atomizer.

## 3. Experimental Methods and Results

Following a general description of PSL particle sizing with the classifier, the measurement methods for defining the accuracy of particle size measurements by the electrical mobility classifier are presented. This section includes a detailed uncertainty analysis of the classifier performance and an analysis of the effect of non-volatile impurities on the PSL sphere size as an aerosol.

### 3.1 Procedure for Sizing Particles with the Electrostatic Classifier

Sizing the PSL spheres with the electrostatic classifier is a relatively fast process. A suspension of particles is prepared, the PSL-particle aerosol is generated, the classifier is used to measure the voltage corresponding to the mean electrical mobility of the PSL spheres, and a straight forward data reduction process provides a measurement of the mean particle diameter. From start to finish, the sizing process takes about 15 min. The liquid suspension of PSL spheres was prepared by diluting a concentrated suspension with deionized-filtered water. The concentrated suspension of the 0.1 μm PSL spheres consisted of about 10% by weight PSL spheres suspended in water. The Standard Reference Material particles, 0.3 and 0.9 μm, were supplied in a suspension of 0.5% by weight PSL spheres in water. The nominal dilutions and particle concentrations of the PSL suspensions used in the atomizer are as follows:

**Table:** 

Particle diameter	Drops of concentrated PSL suspension	dilution volume	concentration #/ml
0.1 μm	3 of 10% by weight	250 ml	6×10^10^
0.3 μm	3 of 0.5% by weight	25 ml	2×10^9^
0.9 μm	10 of 0.5% by weight	25 ml	2×10^8^

While the 0.3 μm and 0.9 μm particle concentrations in the liquid suspension were lower than the 0.1 μm particle concentration, the monodisperse aerosol concentrations were similar. The lower liquid concentrations of the 0.3 μm and 0.9 μm particles is offset by atomizing the suspensions without the impactor. The atomizer produces more particle-carrying droplets without the impactor.

Following a warm-up period to allow the classifier flowmeters to stabilize, the aerosol was generated and passed through the classifier. During normal operafion, the sheath and excess air flow rates were kept equal, resulting in equal polydisperse and monodisperse aerosol flow rates. To minimize the internal pressure of the classifier, the excess air and monodisperse aerosol valves were operated in a fully open position. The sheath air flow rate was set by iterating with the two valves upstream of the sheath air inlet, shown in figure 1, until the pressure between the valves was about 1.60×10^4^ Pa (160 cm water) while maintaining the desired flow rate. The pressure upstream of the sheath air inlet was maintained at 1.60×10^4^ Pa to match the conditions existing during flowmeter calibration. The excess polydisperse aerosol valve and the excess monodisperse aerosol valve were iteratively adjusted to provide the correct excess air and monodisperse aerosol flow rates. The flow rates used for sizing the 0.1 μm particles were nominally 333 cm^3^/s (20 L/min) sheath air flows, and 33.3 cm^3^/s (2 L/min) aerosol flows. For sizing the 0.3 μm particles, the flow rates were nominally 167 cm^3^/s (10 L/min) sheath flows, and 16.7 cm^3^/s (1 L/min) aerosol flows. The 0.9 μm particles were sized using nominally 50 cm^3^/s (3 L/min) sheath, and 5 cm^3^/s (0.3 L/min) aerosol flow rates. Other flow rates were used to investigate the effect of flow rate on size measurements.

Once the flow rates in the classifier were established, the center rod voltage was varied to find the peak in the mobility distribution as measured by the condensation nucleus counter. The concentration was then monitored for several minutes to insure a constant aerosol concentration. The fluctuations in the particle concentration were consistent with a Poisson distribution of number concentration; that is, the coefficient of variation, *CV*, defined as the ratio of the standard deviation in the number concentrafion to the average number concentration, was in agreement with the predicted *CV* for a Poisson distribution.

$$CV=\frac{1}{\sqrt{N}}$$

where

*CV* = coefficient of variation for a Poisson distribution
*N* = average aerosol number concentration.

The concentration recorded for a given voltage setting was obtained by monitoring several consecutive concentration readings (one reading every 3 s) and then estimating the average concentration. When the concentration fluctuations were obviously larger than statistically predicted, the measurement was discarded and efforts were made to stabilize the concentration. Gradual concentration changes over the course of measuring the mobility distribution resulted in slight sizing uncertainties and are included in the estimate of particle diameter measurement uncertainty.

**Figure 7:**
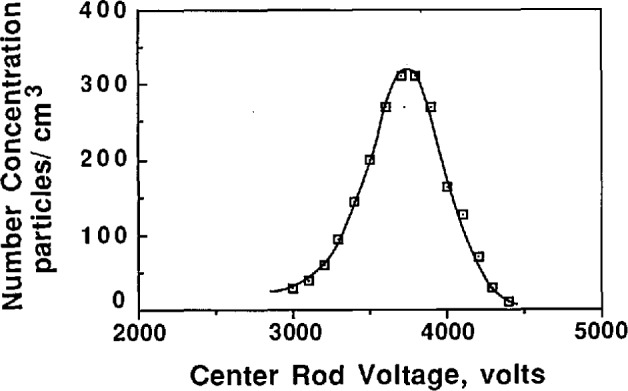
Number concentration vs center rod voltage for 0.1 μm PSL splieres.

After the aerosol concentration stabilized, the voltage on the center rod of the classifier was adjusted symmetrically about the peak concentration voltage. The concentration was recorded for each voltage setting. A typical concentration-voltage curve is shown in figure 7. The quantity of primary interest in this study is the peak voltage which is the voltage corresponding to the peak in the concentration-voltage curve. The peak voltage is computed as the concentration weighted average as follows:

$$V_{\text{ave}}=\frac{\sum V_{i}N_{i}}{\sum N_{i}}$$ (16)

where

*V*_ave_ = peak voltage
*V_i_* = measurement voltages
*N_i_* = concentration corresponding to *V_i_*.

Once the representative voltage of the peak is found, the particle diameter can be calculated using eqs (1) and (12). Since the particle slip correction is dependent on particle diameter, it is necessary to iterate with eq (12) to determine the diameter. A simple iteration routine is used for this purpose.

The calculation of particle diameter from eqs (1) and (12), requires accurate values for the viscosity of air, μ, and the particle slip correction, *C* The slip correction used in the diameter calculations is based on Allen and Raabe’s [14] measurements for PSL spheres using an improved Millikan apparatus:

$$C=1+Kn\left[1.142+0.558\;\exp\left(\frac{-0.999}{Kn}\right)\right]$$ (17)

where

*C* = particle slip correction
*Kn* = Knudsen number
*Kn* = $\frac{2\lambda }{D_{p}}$, where *D*_p_ is the particle diameter
λ = mean free path of air.

Pressure and temperature corrections were made to the mean free path (λ) [15]:

$$\lambda =\lambda_{0}\left(\frac{T}{T_{0}}\right)\left(\frac{P_{0}}{P}\right)\left(\frac{1+\frac{110.4}{T_{0}}}{1+\frac{110.4}{T}}\right)$$ (18)

where

*λ*_0_ = 0.0673 μm, for air at *T*_0_, *P*_0_
*T*_0_ = reference temperature, 296.15 K
*P*_0_ = reference pressure, 1.01×10^5^ Pa (760 mm Hg)
*T* = air temperature; Kelvin
*P* = air pressure inside the classifier.

The coefficient of viscosity of air was calculated as [15]:

$$\mu =\mu_{23{}^{\circ}C}{\left(\frac{T}{296.15}\right)}^{1.5}\left(\frac{286.15+110.4}{T+110.4}\right)$$ (19)

whereμ_23°C_ = 1.93245×10^−4^*P*.

### 3.2 Verification of Correct Classifier Performance

To ensure correct operation of the electrostatic classifier, calibrations were performed and performance tests were conducted. The voltage and flow meters were calibrated, the effect of humidity on volumetric flow rate was measured, the transfer function of the classifier was measured and compared to the theoretically predicted transfer function. A sensitivity analysis was performed to verify the theoretical relationships describing the classifier’s dependence on the operating pressure and the flow rate. The repeatability of size measurements was checked and the classifier measurements were tested with Standard Reference Material particles.

#### 3.2.1 Calibration of the Flowmeters and Voltage Meter

##### Voltage Meter Calibration

The center rod voltage meter was calibrated with a precision voltage meter capable of reading voltages up to 10,000 V. The accuracy of the calibrating meter is estimated as ±0.2%. The calibration was accomplished by connecting the calibrating voltage meter to the lead from the power supply. The center rod voltage meter was calibrated from 1000 to 9,000 V. The calibration indicated that the center rod voltage meter was indicating voltages higher than were actually present by about 2% at 4,000 and 3% at 9,000 V. For sizing 0.1 μm particles using 333 cm^3^/s sheath air, a 2% error in voltage at the nominal voltage peak of 3,800 V corresponds to a 1% error in particle diameter. For sizing 0.3 μm particles using sheath air at about 167 cm^3^/s, a 3% error in voltage at a nominal voltage peak of 8,000 V corresponds to a 2% error in diameter.

##### Flowmeter Calibration

Calibrations of the mass flowmeters used to control sheath air flow, excess air flow, and monodisperse air flow were performed to improve the accuracy of the size measurement. The calibrations were performed at the NIST flow calibration facility using the “piston prover” apparatus, maintained as the primary standard for calibration of gas flow meters. The apparatus consists of a volume displacement device incorporating a mercury-sealed piston inside of a glass cylinder. For calibration of a flow meter, dry gas is passed through the meter and into the calibration cylinder. The piston is displaced through an accurately defined volume in an accurately measured time. A bypass valve allows re-routing of the gas stream so the piston may be returned to its original configuration between each calibration run. Measurements of temperature and pressure are recorded so that the mass flow rate can be determined.

In order to eliminate changes in the flowfield experienced by the flowmeters, the calibrations were conducted without removing the flowmeters from the classifier. The configuration of the classifier allowed simultaneous calibration of either the sheath air meter and the excess air meter, or the sheath air meter and the monodisperse aerosol meter. To calibrate the sheath air meter and the excess air meter, the monodisperse aerosol outlet valve was closed and the polydisperse aerosol inlet was plugged. To calibrate the sheath air meter and the monodisperse aerosol meter, the polydisperse aerosol inlet was left plugged, the excess air valve was closed, and the monodisperse aerosol valve was left fully open.

The calibrations were performed with the classifier valves in their normal configuration (excess air and monodisperse aerosol valves fully open). The valve on the sheath air inlet was adjusted to provide an upstream air pressure of about 1.60×10^4^ Pa, and this pressure was maintained during normal operation of the classifier.

During calibration, the flow rate was approximately selected using the manufacturer’s original calibration. The meter readings were recorded, and the flow rate was measured using the “piston-prover” calibration apparatus described above. The flow rates chosen for calibration of the flowmeters were nominally 333, 167, and 50 cm^3^/s for the sheath air and excess air flow rates. These flow rates were chosen to maximize the flow accuracy for sizing 0.1 μm, 0.3 μm SRM, and 0.9 μm SRM particles, respectively. For the monodisperse aerosol flow meter, the calibration flow rates ranged from 33.3 to 4.2 cm^3^/s. The flow meters were calibrated at additional flow rates in the vicinity of the nominal values listed above.

Each calibration point was repeated five times on two consecutive days, and a partial calibration was conducted 1 week later to check for meter drift. The accuracy which is normally quoted by the NIST calibration facility is on the order of ±0.25%, with 99% confidence. As will be discussed later, the estimate of uncertainty in the flowmeters used during operation of the classifier is conservatively estimated to be ±1% due to additional uncertainties in the meter setting and the temperature and pressure which are used to convert the mass flow rate to the volumetric flow rate [eq (15)]. The calibration conducted a week after the initial calibration did not indicate a significant drift for the higher flow rate calibrations (maximum shifts for sheath and excess air: 0.05% at 33.3 cm^3^/s 0.14% at 167 cm^3^/s, and .01% at 333 cm^3^/s). Drift associated with the monodisperse aerosol meter using lower flow rates was slightly higher, with the maximum shift between the three calibration days of about 0.5% for flow rate settings of nominally 33.3, 16.7, and 4.2 cm^3^/s.

The manufacturer’s calibration for the sheath air meter indicated lower flow rates by about 5% at nominally 333 cm^3^/s and 3% at nominally 167 cm^3^/s compared to the NIST flow rate calibration. The manufacturer’s calibration for the excess air meter was found to be 8% lower at nominally 333 cm^3^/s and 7% lower at nominally 167 cm^3^/s. For sizing 0.1 μm particles, an error in the sheath air of 5% at 333 cm^3^/s corresponds to a diameter error of about 3%. It should be noted that although the electrostatic classifier was not used until initiation of this project in 1988, the calibration is dated 7/83. Also, the larger uncertainties seen in the excess air meter may be due to the uncertainty in the meter setting caused by a significant amount of rapid fluctuation in the meter reading.

#### 3.2.2 Effect of Humidity on the Volumetric Flow Rate

Since the molecular weight of a water molecule is less than the molecular weight of air, for a given mass flow rate, the equivalent volumetric flow rate of wet air should be higher than the volumetric flow rate of dry air. Water vapor, produced by the atomizer, leads to high humidities of the air exiting the atomizer. While the drying tube and dilution of the atomizer aerosol with dry air reduces the humidity of aerosol entering the classifier, the resulting air humidity is still higher than the humidity of the air used during flowmeter calibration. The air used for sheath air is sent through a diffusion dryer providing relative humidities on the order of 5%. The relative humidity of the aerosol at the classifier inlet can be high if low dilution air is used. The flow rate from the atomizer without dilution is 83.3 cm^3^/s, and a typical dilution air flow is about 80 cm^3^/s. When the atomizer was used with the impactor, the relative humidity of the aerosol at the inlet to the classifier was measured to be around 25% and about 7% at the excess air outlet.

For wet air, the volumetric flow rate correction made to the flowmeter calibration is slightly different from the correction made for dry air. Assuming flowmeter calibrations are conducted with dry air, the volumetric flow rate correction for wet air, derived based on ideal gas considerations, is as follows [compare to eq (15)]:

$$Q_{\text{vol}}=Q_{\text{cal}}\cdot \frac{T_{\text{actual}}}{T_{\text{cal}}}\cdot \frac{P_{\text{cal}}}{P_{\text{air}}+P_{H_{2}O}\frac{M_{H_{2}O}}{M_{\text{air}}}}$$ (20)

*Q*_vol_ = volumetric flow rate of wet air
*Q*_cal_ = calibrated volumetric flow rate at *T*_cal_,*P*_cal_
*P*_air_ = partial pressure of air
$P_{H_{2}O}$ = partial pressure of water vapor
$M_{H_{2}O}$ = molecular weight of water
*M*_air_ = molecular weight of air.

The effect of relative humidity on volumetric flow rate predicted by eq (20) is summarized below:

**Table:** 

R.H. (%)	*Q*_vol_ with R.H. correction	
	*Q*_vol_ no R.H. correction	
0	1.000	
10	1.001	(0.1%)
20	1.002	(0.2%)
50	1.006	(0.6%)
70	1.009	(0.9%)
100	1.012	(1.2%)

Typical relative humidities of the excess air measured when sizing 0.1 μm particles were 5–15%. The relative humidities existing when sizing the 0.3 and 0.9 μm SRM particles were higher (when the atomizer is used without the impactor, more water vapor is produced). The humidities were not measured in these cases; however, an upperbound humidity of 25% is estimated based on 100% humidity of the inlet aerosol and a factor of 10 dilution by the dry sheath air. Particle diameter measurements made without correcting the flow rate for relative humidity will result in an increase in the measured diameter by a magnitude approximately half the flow volume ratios shown above.

The effect of humidity on the volumetric flow rate was experimentally investigated using a gas-test meter. Maintaining a given voltage on the mass flowmeter, the volumetric flow rates were measured with different air humidities. It was found that changing the humidity from 5% to 60% for fixed mass flow rate increased the volumetric flow rate by less than 0.5%, which was at the resolution limit of the flow measurement. This finding is consistent with eq (20), but the measurement resolution is inadequate to provide a quantitative test of the equation. In any event for the 0.1 μm PSL spheres with a humidity of 5–15%, the predicted humidity correction to the volumetric flow rate is less than 0.2%.

#### 3.2.3 Testing the Transfer Function

To determine whether the classifier is operating correctly, its performance can be judged by comparing the theoretical and experimental output of the classifier when classifying a monodisperse aerosol. Figure 8a shows a plot of concentration vs center rod voltage for 0.269 μm PSL under the condition of equal aerosol flow rates. Also shown on the curve is the theoretical voltage-concentration curve plotted about the peak concentration voltage, which was obtained from figure 5 and eq (11) with *Q*_c_ = *Q*_m_ = 167 cm^3^/s and *Q*_a_ = *Q*_s_ = 17 cm^3^/s. While Figure 8a indicates approximately correct behavior, the slight uncertainty in the peak concentration causes uncertainty in the placement of the theoretical transfer function. This uncertainty is the result of the rounding effect at the peak caused by slightly unequal aerosol flow rates. A better comparison is obtained if the aerosol flow rates are not equal. The flow rate of the aerosol entering the classifier was reduced by a factor of two, *Q*_a_ = 8.5 cm^3^/s, while the sheath flow was increased by 8.5 cm^3^/s so *Q*_c_ = 175 cm^3^/s (fig. 8b). The data in figure 8b allows definite placement of the theoretical transfer function and indicates correct classifier output. Slight differences between the theoretical transfer function and the experimental transfer function are due in part to the fact that the PSL is not perfectly monodisperse.

A second method to check for correct performance of the classifier was developed by Rader and McMurry [9] and involves the use of two classifiers in series. Such a configuration is called a TDMA (Tandem Differential Mobility Analyzer). In this method, a polydisperse aerosol is sent through the first classifier to produce a test aerosol for the second classifier. The voltage on the first classifier is held constant while the voltage on the second classifieris varied to trace the distribution of the test aerosol. The concentration-voltage data of the second classifier is then compared to the TDMA theory using a computer algorithm which estimates the ratio of sheath to aerosol flow rates by fitting the theoretical relationships to the voltage-concentration data. Agreement between the actual flow ratio and the fitted flow ratio is an indication that the classifiers are operating correctly. This method was used to test the performance of the NIST classifier using a second classifier of the same type and model to complete the TDMA system. The second classifier was provided by the University of Minnesota Particle Technology Lab, The results of the TDMA test indicated the classifier was operating correctly. (For a sheath to aerosol flow ratio of 10.0, the algorithm indicated a ratio of 9.8 with the NIST classifier used as the second classifier in the TDMA system, and a ratio of 10.0 with the NIST classifier used as the first classifier in the TDMA system.)

A third test of the classifier’s performance is to compare the experimental peak concentration at the output of the second classifier (*N*_2out_) to the concentration at the input to the second classifier (*N*_in_). From the triangular shape of the inlet mobility distribution function (see fig. 5) and from a similar triangular shape for the sampling efficiency of the second classifier, Kousaka et al. [7] derived the following relationship between *N*_2out_ and *N*_in_ for the case where the voltage of the second DMA is set equal to the first:

$$\underset{\left(\text{theoretical}\right)}{N_{2\text{out}}}=\left(\frac{2}{3}\right)N_{\text{in}}.$$ (21)

The following experimental results indicate again that the classifier performs as predicted:

**Table:** 

*N*_2out_	*N*_in_	$\underset{\left(\text{theoretical}\right)}{N_{2\text{out}}}=\left(\frac{2}{3}\right)N_{\text{in}}$	*N*_2out_
			$\underset{\left(\text{theoretical}\right)}{N_{2\text{out}}}$
335	540	360	0.93
445	730	486	0.92

The 7% to 8% difference between the actual and theoretical downstream concentrations is due to particle wall losses within the second DMA and to slight differences between the actual and theoretical transfer functions.

#### 3.2.4 Sensitivity Analysis

To investigate the equations governing the size measurement of the PSL spheres, the operating conditions of the classifier were varied slightly and then the PSL spheres were sized. If the equations governing particle size measurement are correct, the measured particle diameter should remain the same regardless of which operating conditions are used.

The experimental method was straightforward. The variables which lend themselves to variation are flow rate and operating pressure. The flow rate affects the relationship between the center rod voltage and the particle electrical mobility [eq (12)], and the pressure effects the volumetric flow rate [eq (15)] and the particle slip correction [eq (17)] through its effect on the mean-free path of air [eq (18)]. The measurement consisted of first sizing 0.1 μm PSL using nominally 333 cm^3^/s sheath flow and excess flow rates, and a normal operating pressure of approximately 3.5×10^3^ Pa above ambient. The particle diameter measured with these operating conditions was compared to the diameter measured when the flow rate or pressure were changed. Equivalently, the governing equations can be used to predict the change in the peak voltage which should result when a different flow rate or pressure is used for the measurement. The predicted peak voltage can be compared to the experimentally measured peak voltage.

##### Operating Pressure Variation

The measurement using different operating pressures was done by restricting the excess air valve so that the pressure inside the classifier increased from the normal operating pressure of 3.5×10^3^ Pa above ambient to about 1.27×10^4^ Pa above ambient. The increase in pressure results in a decrease in the volumetric flow rates [eq (15)], and a decrease in the particle slip correction factor [eq (17)]. The governing equations [eqs (1) and (12) together with the expression for the slip correction] predict that the increase in pressure should result in a decrease in the peak voltage of about 3%, which was within 0.2% of the measured decrease in the peak voltage, 3780 to 3685 V. The particle size measured with a pressure of 1.27×10^4^ Pa was within 0.1% of the size measured using the normal pressure of 3.5×10^3^ Pa. The agreement between the two measurements of particle diameter and the agreement between the predicted and measured change in the peak voltage indicate that the pressure variable is incorporated correctly into the governing equations of the particle measurement.

##### Sheath Flow Variation

The sheath flow rate was decreased from 333 to 300 cm^3^/s while keeping the excess air flow rate at 333 cm^3^/s. To maintain a flow balance, the polydisperse aerosol flow rate was operated at 66 cm^3^/s, while the monodisperse aerosol flow rate was operated at 33 cm^3^/s. In this case, the governing equations predict a decrease in the peak voltage of about 6% which was within 0.6% of the measured change in peak voltage, 4240 to 4000 V. The difference between the particle size measured using the normal operating conditions and the varied-flow rate conditions was less than 0.3%. This difference is probably caused by a dependence of measured particle diameter on the aerosol flow rate, which is discussed below.

##### Aerosol Flow Variation

Particle sizing was also conducted using different aerosol flow rates. In this case, the theory predicts a change in the mobility width of the monodisperse aerosol outlet particles [eq (13)], but does not predict a change in the mean electrical mobility or measured particle diameter. A high aerosol flow rate corresponds to a wide electrical mobility range of the particles sampled through the slit in the center rod. A low aerosol flow rate corresponds to a narrow electrical mobility range of the monodisperse aerosol outlet particles.

To study the effect of the aerosol flow rate on particle size, the sheath flow and excess flow rates were kept constant and equal while the two aerosol flow rates were varied in tandem. Figures 9 and 10 show the effect of aerosol flow rate on the voltage-concentration curve for 0.269 μm and 0.1 μm PSL spheres. The sheath flow and excess flow rates were 167 cm^3^/s for the 0.269 μm particles and 333 cm^3^/s for the 0.1 μm particles. Figures 9 and 10 show the distribution plotted with both actual concentration and normalized concentration. Plotting normalized concentration allows direct comparison of the peak voltage. For the 0.1 μm distribution, the particle diameter increases about 1% as the aerosol flow rate was decreased from 33.3 to 5.0 cm^3^/s. The voltage peak was determined by averaging the concentration-voltage data, using eq (16), for concentrations greater than 0.6 *N*_max_. For the 0.269 μm PSL spheres, an increase in diameter of about 1% was also found for decreasing aerosol flow rates.

The reason for this increase in particle size is not presently known. The slight sizing dependence on aerosol flow rate is negligible for typical applications of the electrostatic classifier. This effect apparently has not been reported in the literature. For this work, the increase in measured diameter for decreasing aerosol flow rates is included as an uncertainty in the measured diameter.

#### 3.2.5 Sizing Repeatability

The 0.1 μm PSL size measurement was repeated eight times on one day and six times about a week later. The 14 measurements are shown in table 1. The sheath flow and excess flow rates used for these measurements were 340 and 330 cm^3^/s for the first and second days, respectively, while aerosol flow rates were nominally 33 cm^3^/s. The coefficient of variation (*CV*) of the 14 measurements is 0.2%. The size measurements for runs 1–3 on day 1 are thought to have been affected by a gradually changing inlet concentration. If runs 1–3 on day 1 are discarded, the *CV* of the measurements is 0.1%.

#### 3.2.6 Measurement of Standard Reference Material Particles

As a test of the sizing accuracy of the classifier. Standard Reference Material particles (NIST SRM 1691 at 0.269 ± .007 μm, and NIST SRM 1690 at 0.895 ± .008 μm) were sized. The resulting size measurements are shown in table 2. The measurements were made immediately following the flowmeter calibration and include the voltage calibration. The 0.269 μm SRM particles were measured using sheath flow and excess flow rates of 167 cm^3^/s and aerosol flow rates of 17 cm^3^/s. The 0.895 μm SRM particles were measured using sheath flow and excess flow rates of 41.7 cm^3^/s and aerosol flow rates of nominally 5 cm^3^/s. The 0.269 μm SRM particles, measured for the SRM report using electron microscopy, were measured with the classifier to have a mean diameter of 0.273 μm, which is 1.6% larger than the SRM reported diameter. The uncertainty in the diameter of the 0.269 μm particles is 2.6%. The 0.895 μm SRM particles, measured for the SRM report using a light scattering technique, were measured with the classifier to have a mean diameter of 0.910 μm, which is 1.7% larger than the SRM reported diameter. The uncertainty in the diameter of the 0.895 μm particles is ±0.9%.

While both measurements are larger than the SRM reported diameters by a similar percentage, the measurement of the 0.269 μm particles lies within the uncertainty quoted for the SRM measurement, but the measurement of the 0.895 μm SRM particles is outside of the error band quoted for the SRM measurement. It should be noted that the measurement of the 0.895 μm SRM particles was conducted using significantly different flow conditions (41.7 cm^3^/s) than those used for the 0.1 and 0.269 μm SRM particles (333 and 167 cm^3^/s sheath flows, respectively). The 0.895 μm particle measurement can be repeated using higher sheath flows by measuring multiply charged particles. This method is described below in section 3.2.7.

#### 3.2.7 Calibration of the Electrostatic Classifier Using Standard Reference Material Particles

One possible explanation for the difference between the electrical mobility results for particle size and the certified particle size is an error in the definition of the length of the classifier. Recall that the length dimension is used in eq (12) to calculate the particle electrical mobility from which the particle size is calculated using eq (1). At present, the length is defined as the distance from the midpoint of the monodisperse aerosol exit slit to the midpoint of the aerosol inlet (see fig. 3). This choice of length is consistent with the analysis by Knutson and Whitby [1] assuming axisymmetric and laminar flow and a uniform electric field in the axial direction. These conditions will be violated to some extent at the aerosol entrance and exit to the classifying column. These effects might be incorporated in eq (12) as a corrected length of the classifier. If the length used in the calculations is taken as 1.9% shorter than the presently defined length, (44.44 cm changed to 43.60 cm), the classifier measurements of both SRM particle sizes agree within 0.1% with the SRM reported diameters. The length dimension was measured in this study to be 44.37 cm which is in close agreement with the 44.44 cm measurement reported by the manufacturer. While the length can be adjusted so the classifier indicates the correct size for both SRM particle sizes, the required change in length may be too large to claim that the measurement differences are due to an error in the length definition.

Adjusting the length definition as suggested above is one method of calibrating the electrostatic classifier for measurement of the 0.1 μm particles. A more rigorous approach for calibrating the classifier, which is suggested for future consideration, is to measure the 0.269 and 0.895 μm SRM particles using the same sheath and aerosol flow rates as used for the measurement of 0.1 μm particles. The calibration technique involves measuring the electrical mobility of multiply charged 0.269 and 0.895 μm SRM particles. Since a multiply charged particle has a higher electrical mobility than a singly charged particle, a higher flow rate can be used in the classifier to measure the mean particle mobility and particle diameter. By measuring the multiply charged SRM particles with the same flow conditions as the 0.1 μm particles, a calibration factor (such as changing the length definition) can be included in the governing equations which forces the SRM particle measurements to be in agreement with the reported diameters. This method of calibration is thought to be more rigorous since all the particles are measured with the same flow conditions.

### 3.3 Investigating the Effect of Impurities

As was discussed in section 2.1, impurities in the water used to suspend the PSL spheres produce a layer of residue on the surface of particles after the PSL-carrying atomizer droplets evaporate. This residue results in a systematic error in particle diameter measurements since it increases the apparent particle diameter. The impurities in the PSL particle suspension come from impurities existing in the water used to dilute the concentrated PSL particle suspension and from the impurities in the liquid used in the concentrated PSL particle suspension. To estimate the thickness of the impurity residue on the PSL sphere, it is necessary to know the impurity concentration in the PSL particle suspension and the diameter of the particle-carrying droplet. Assuming all of the non-volatile impurity forms a uniform residue shell around the particle, the following relationship between the thickness of the residue on the particle and the impurity concentration, particle diameter, and droplet diameter is obtained:

$$t=\frac{C{\left(D_{d}\right)}^{3}}{3{\left(D_{p}\right)}^{2}}$$ (22)

where

*t* = impurity addition to diameter (μm)
*C* = volumetric concentration of impurities
*D*_d_ = PSL-carrying droplet diameter before evaporation (μm)
*D*_p_ = PSL particle diameter (μm).

To estimate the effect of impurities on the particle size, measurements were performed to determine the PSL particle-carrying droplet diameter before evaporation, the concentration of impurities in the water used to dilute the PSL particle suspension, the impurities in the diluted PSL particle suspension, and the impurity concentration effect on particle diameter.

#### 3.3.1 Characterizing the Atomizer

As seen in eq (22), the PSL-carrying droplet diameter strongly influences the effect of impurities on particle diameter. The droplet distribution was determined by atomizing a solution containing a known concentration of NaCl, and measuring the resulting residue particle size distribution using the classifier. The droplet size distribution can then be determined using the simple relationship between the impurity concentration, *C*, residue particle size, *D*_p_, and the droplet size, *D*_d_:

$$D_{p}=D_{d}\cdot C^{\left(1/3\right)}.$$ (23)

The atomizer can be used in two configurations. First, for sizing the 0.1 μm particles, an impactor is used at the outlet of the atomizer to remove large droplets (see fig. 2). Without the impactor the larger droplets, capable of carrying larger PSL spheres, are allowed to pass through the outlet of the atomizer. Figure 11 shows the number distribution of droplets produced with and without the impactor. With the impactor in place, the mode of the number distribution is around 0.7 μm. Without the impactor, the mode of the number distribution is around 0.8 μm, with significantly more large droplets than exist with the impactor. The effect of the impactor is more obvious if the droplet distribution is weighted by mass or volume as shown in figure 12.

Similar measurements of an atomizer’s droplet distribution using this technique, reported by Niida et al. [16], suggest that the droplet distributions measured in this work are biased toward larger particles because of diffusional losses of small residue particles upstream of the classifier. The measured droplet distributions are only qualitatively representative of the actual distributions. The droplet distribution of the atomizer suggests that sizing PSL spheres without the impactor in place will result in larger PSL particle-carrying droplets, and more significant impurity effects.

#### 3.3.2 Measuring the Concentration of Impurities in the Water Used to Dilute the PSL Particle Suspension

The volumetric impurity concentration was measured for tap water and distilled, deionized water using three methods. The tap water impurity concentration measurements were conducted for comparative purposes. Two measurement methods involved evaporating droplets and measuring the resulting residue particle size. The third method involved gravimetric measurements of evaporation residue.

##### Classifying Atomized DI Water

In the first method used to estimate water impurities, the water was atomized without PSL or other additives. The atomizer was used without the impactor so that larger residue particles were formed. The droplets formed from the atomization were dried and the resulting residue particles were sized using the classifier. This measurement was done for tap water and distilled, deionized (DI) water, and the resulting mass distributions are shown in figures 13a and 13b along with the residue particle distributions produced by atomizing a known solution of NaCl.

The calculation of impurity concentration is accomplished by comparing the means of the mass distributions of the water residue and NaCl residue particles. The DI water residue particles were compared to the residue particles produced from a solution of 0.0052% NaCl by volume. The tap water residue particles were compared to the residue particles produced from solution of 0.0108% NaCl by volume. The impurity concentrations were calculated from the following expression derived from eq (22):

$$C_{\text{water}}={\left(\frac{D_{p\;\text{NaCl}\;\text{mode}}}{D_{p\;\text{water}\;\text{mode}}}\right)}^{3}\times C_{\text{NaCl}}$$ (24)

*C*_water_ = volumetric impurity concentration in water
*C*_NaCl_ = volumetric concentration of NaCl
*D*_p NaCl mode_ = mode of the NaCl residue distribution
*D*_p water mode_ = mode of the water residue distribution.

The result of the impurity measurement follows from figures 13a and 13b:

**Table:** 

Atomizer solution	Residue particle mode (μm)	Volumetric impurity concentration	Uncertainty
0.0052% NaCl	0.11	0.0052% (52 ppm)	
Dl-water	0.04	0.0002% (2 ppm)	± 1 ppm^a^
0.0108% NaCl	0.15	0.0108% (108 ppm)	
Tap water	0.20	0.026% (260 ppm)	± 70 ppm

a Uncertainties resulting from estimation of mode diameter.

One problem with sizing the residue particles from the DI water is the loss of particles downstream of the atomizer before being classified. To reduce electrostatic losses a Kr-85 bipolar charge neutralizer was added at the outlet of the atomizer for the DI water residue particles and the 0.0052% NaCl residue particles. Evidence of these losses is apparent from the observation that the number concentration of residue particles for the 0.0052% NaCl solution is about 30 times greater than the concentration of the residue particles produced from the DI water. This would suggest that the mode of the number distribution of the residue particles from the DI water is outside the range of the classifier. The mode of the mass distribution of the residue particles appears to be within the range of the classifier.

##### Classifying Residue Particles From the Vibrating Orifice

The second method used to determine the volumetric concentration of the impurity in the water again involved sizing the residue particles produced by evaporating large water droplets of known size. In this method the Vibrating Orifice Monodisperse Aerosol Generator (VOAG) (TSI, Inc., Model 3450) was used to produce large monodisperse water droplets. The vibrating orifice generator was operated without a filter on the liquid pump so that impurities were not removed from the solution being tested. The VOAG was used to produce 39 μm droplets of the DI water. The resulting residue particles were sized with the classifier. Except for a secondary peak corresponding to doubly-charged particles, the residue particles were monodisperse with a size of about 0.27 μm. Using eq (22), the concentration of impurities in the water is calculated to be 0.3 ppm.

**Figure 14:**
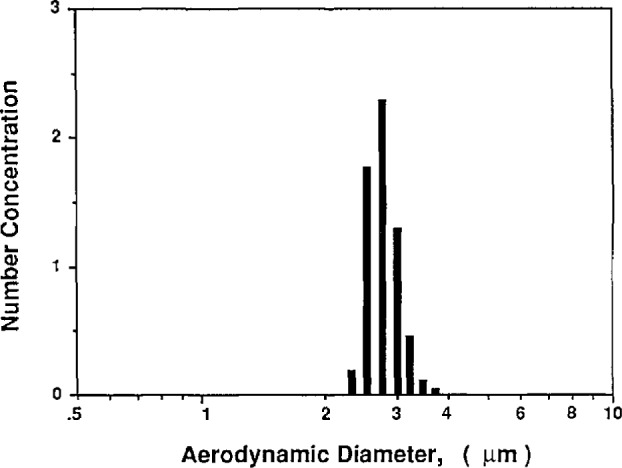
Tap water residue particles produced by the vibrating orifice and measured with the aerodynamic particle sizer.

This same method was used to estimate the level of impurities in normal tap water and lab distilled water. Using the classifier to size the residue particles for normal distilled water, the impurity concentration was calculated to be 5 ppm. For normal tap water, the residual particles were too large to size using the classifier. Instead the TSI Model 3310 Aerodynamic Particle Sizer (APS) was used. The resulting distribution is shown in figure 14 to have a peak particle aerodynamic diameter of about 2.7 μm. For a unit density particle, the aerodynamic diameter is equal to the geometric diameter, and the volumetric impurity concentration can be estimated using eq (22) to be 330 ppm.

##### Impurity Measurements using a Gravimetric Method

A third attempt at measuring the volumetric impurity concentration of the water was to evaporate a known mass of water and measure the resulting impurity mass. This method did not work for the distilled water or the DI water because the impurity mass was too low. The method did work for the tap water and indicated a mass concentration of about 220 ppm.

The three methods for measuring the impurity concentration are qualitatively in agreement. For the deionized/filtered water, the methods indicated impurity concentrations on the order of 1 ppm. The impurity concentration measurements of the tap water were conducted to compare the different methods. The measurements made with the VOAG-APS and the gravimetric method are in qualitative agreement, with best agreement occurring if unit density impurity is assumed. For unit density impurity, the VOAG-APS indicates 330 ppm volumetric impurity and the gravimetric method indicating 220 ppm volumetric impurity. As stated above, the tap water impurity was measured by sizing residue particles from the atomizer to be about 260 ppm.

#### 3.3.3 Estimating the Impurity Concentration in the PSL Particle Suspension

In the previous section, the impurity concentration in the water used to dilute.the PSL suspension was measured. The second source of impurities, the concentrated PSL suspension, is considered below. The total non-volatile impurity concentration in the diluted suspension is estimated in this section and also the predicted effect of the impurity on the particle diameter is compared with measurements.

##### Calculation of the Impurity Concentration

Concentrations of impurities in the solids composing PSL particle suspensions have been reported to be from about 1 to 7% [17]. If the concentrated PSL particle suspension is not sufficiently diluted with water, these impurities will have a significant effect on the resulting particle size. For the 0.1 μm particles, the percentage of solids in the concentrated PSL suspension was about 10%. Dilution of the concentrated suspension was normally about 1 to 2500 with DI water. Assuming an impurity concentration in the PSL solids to be 3% (in the middle of the range reported by Raabe [17]), the resulting PSL particle suspension, including 1 ppm impurity in the dilution water, will have an impurity concentration of about 2.2 ppm. Using eq (22) with a droplet diameter of about 0.9 μm, the resulting residue thickness (in diameter) is about 0.00005 μm or about a 0.05% addition to the diameter. The same calculation for the 0.269 μm SRM particles containing 0.5% solids and 50 ppm of biocide in the concentrated PSL particle suspension, (assuming 3% impurities in the PSL solids, a dilution of 1 to 250, and a larger droplet diameter of 2.5 μm since sizing occurs without the impactor on the atomizer), indicates that an increase in diameter of 0.0001 μm or about 0.06% would be expected.

##### Measurement of the Impurity Concentration

**Figure 15:**
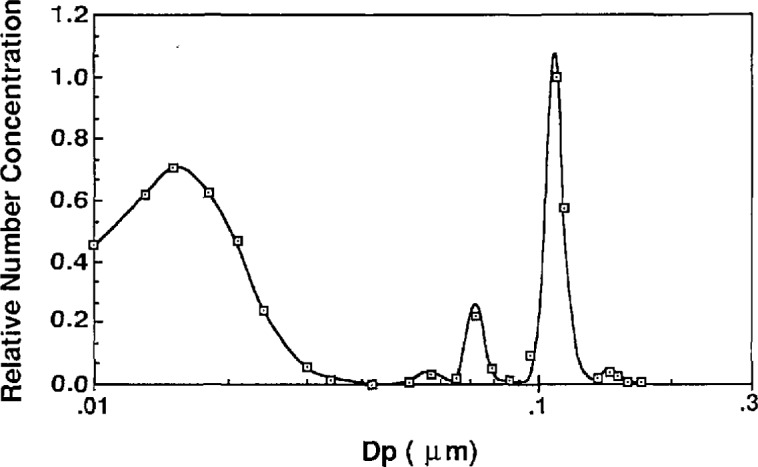
Number concentrations vs particle diameter for 0.1 μm PSL particles and associated impurity residue particles.

Measuring the concentration of impurities in the actual PSL suspension is difficult. As discussed in section 2.1, the aerosol produced by the atomizer consists of both PSL spheres and impurity particles resulting from evaporation of droplets which do not contain a PSL particle. Measurement of the impurity concentration in the PSL suspension was made by classifying the entire distribution of particles existing in the PSL particle aerosol, and comparing the distribution of impurity particles to the distribution of particles produced by atomizing a solution with a known concentration of NaCl. In figure 15, the entire distribution of the 0.1 μm PSL aerosol is shown. The secondary peaks in the vicinity of the main PSL particle peak are the result of doubly charged singlet particles and various multiplet particles. The distribution of impurity particles is clearly identified. The mode of the impurity particle mass distribution was compared to the mode of residue particles produced from a 0.0052% NaCl solution indicating the impurity concentration to be about 6 ppm. This same technique was used to estimate the impurity concentration existing in the 0.269 SRM particles using a typical dilution of the concentrated PSL suspension by about 1 to 300 with DI water. The resulting impurity residue particles were compared to impurity particles from a 0.0108% NaCl solution. The measurement indicated an impurity concentration of about 52 ppm. This result is significantly higher than the estimated concentration using Raabe’s estimates of impurities in the PSL solids.

##### Effect of Impurities on the SRM Particle Measurements

A simple method of determining whether or not the impurities in the PSL solids are contributing to measurement errors is to size the PSL spheres using a very dilute PSL particle suspension, and compare the measurement to that resulting from a very concentrated PSL particle suspension. If the impurities in the undiluted PSL suspension are causing significant measurement errors, the diameter measured using a very dilute suspension should be smaller than the diameter measured using a concentrated suspension. This measurement was conducted for the 0.269 μm SRM particles. The concentration of the PSL suspension was varied by over a factor of 15 (from 3 drops of undiluted PSL in 500 ml of DI water to 10 drops of undiluted PSL in 100 ml of DI water), and no systematic change in the diameter measurement was noticed.

An attempt was also made to size the 0.269 nm SRM particles using an impactor on the atomizer in an effort to reduce the effect of impurities in the suspension. The diameter measured with the impactor was similar to the diameter measured without the impactor giving strong evidence that impurities are not influencing the measurement. It is noted, however, that atomizing the 0.269 μm particles with the impactor leads to extremely low particle concentrations because most of the particles are removed by the impactor. As discussed in section 3.1, if the concentration of the PSL spheres in the aerosol is too low, significant uncertainties result. Based on these two measurements, it is concluded that impurities increase the particle diameter for the 0.269 μm SRM by less than 1%.

##### Effect of Impurities on Measurements of the 0.1 μm Particles

**Figure 16:**
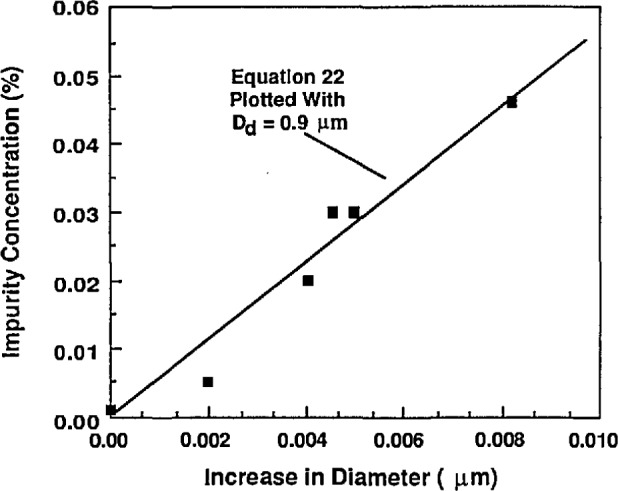
Impurity concentration vs increase in diameter for 0.1 μm particles in a NaCl solution.

To investigate the relationship between impurity concentration and particle size, the nominally 0.1 μm PSL spheres were sized using aqueous NaCl solutions of known concentration. In this measurement, the atomizer was used with the impactor in place because without the impactor the concentration of NaCl particles overshadowed the PSL distribution. The particles were first sized using clean water for suspension and then sized using several different NaCl solutions. Figure 16 summarizes the results. One of the data points used in the figure is the result obtained when the PSL is classified with tap water. Here the impurity concentration of the tap water was 0.033% as measured by sizing the residue particles produced by the Vibrating Orifice Generator with the Aerodynamic Particle Sizer.

This data can be used to predict the effective PSL particle-carrying droplet diameter. The predicted increase in diameter by eq (22) for a droplet diameter of 0.9 μm is seen to be in good agreement with the data in figure 16. This diameter can be used, with an estimate of the impurity concentration in the PSL suspension, to calculate the expected residue thickness added to a particle. Assuming an impurity concentration in the 0.1 μm particle suspension of about 10 ppm as calculated and as measured, eq (22) is used to estimate the impurity residue increase in diameter to be about 0.2%. Several data points taken with NaCl concentrations of about 900 ppm, indicating thicknesses of about 0.01 μm, were not included in Figure 16. This data indicated much lower thickness than would be expected possibly due to clumping of the residue on the surface of the particle. This data also did not agree with droplet distribution data obtained using lower NaCl concentrations, possibly because the NaCl residue particles were forming as hollow clumps.

A final method for estimating the effect of impurities involved sizing of dilute and concentrated PSL suspensions. For PSL concentrations of 3 drops per 1000 ml to 3 drops per 25 ml of DI water, no noticeable size shift occurred. This would suggest that impurities are not influencing the measured diameter of the 0.1 μm particles. If eq (22) is used with the previously estimated impurity concentration of 6 ppm, and particle-carrying droplet diameter of 0.9 μm, the resulting increase in diameter can be estimated to be 0.00015 μm or 0.15%. The uncertainty estimate is approximate and we double the value given above so that the overall uncertainty from impurities is 0.00030 μm or 0.3%.

**Figure 17:**
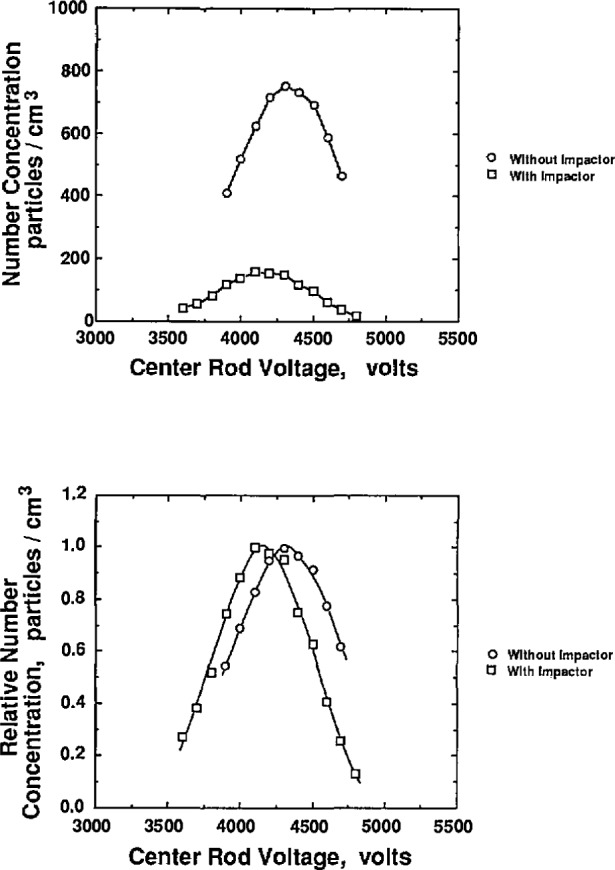
Effect of impactor on particle mobility.

The use of an impactor immediately downstream of the atomizer further reduces the impurity effect by removing the larger droplets. The impactor reduces the peak voltage of the mobility distribution of the 0.1 μm particles by about 130 V as indicated in figure 17. This corresponds to about an 0.002 μm (2%) reduction in the particle size. For the estimated impurity concentration of 6 ppm, a droplet size of 2.2 μm is estimated using eq (22). Thus it is found to be very important to use an impactor to minimize the droplet size in addition to using high purity dilution water.

### 3.4 Estimates of Uncertainty in the Classifier Performance in the Particle Diameter Measurements

In the previous section, results were presented regarding the precision associated with repeat measurements and uncertainties associated with the quantities appearing in the governing equations, eqs (1) and (12), including flow rate, voltage, and slip correction. In this section, a summary is presented of all the uncertainties and an estimate of the overall uncertainty is given for the electrical mobility classifier. An overall estimate of the uncertainty in measuring the 0.1 μm. PSL spheres is obtained by combining the tmcertainty associated with the impurity effect and the uncertainty associated with the use of the classifier.

#### 3.4.1 Random Error

The random component of the uncertainty associated with the measurement of the average particle size can be obtained from the 14 repeat measurements of the particle size (sec. 3.2.5). The average of these 14 measurements and the associated standard deviation, σ, are 0.1068 and 0.0002 μm, respectively. The random component of the uncertainty, *R*, is given by

$$R=t_{n-1}\left(0.025\right)\sigma /{\left(n\right)}^{1/2},$$ (25)

where *n* is the number of repeat measurements, 14, and *t_n_*_−1_ (0.025) is the Student *t*-value for *n*−1 degrees of freedom and for 95% confidence level (*t*_13_ (0.025) = 2.16). The value of *R* is 0.0001 μm, which corresponds to a relative error of 0.1%.

#### 3.4.2 Uncertainty in the Flow Rate

The flow rate uncertainty reported in table 3 represents a combination of flow meter calibration accuracy, precision of flow rate selection, the uncertainties in the pressure and temperature correction to the flowmeter calibration, and the effect of humidity on the flow calibration. The precision of flow rate selection is estimated to be ±0.4% reflecting the stability of the flow rate after it is set, and the precision of the initial flow rate setting. This value was calculated for the sheath air meter at nominally 333 cm^3^/s by estimating the precision of flowmeter voltage setting to be ±.002 V for a voltage setting of 3.160 V. The uncertainty in voltage was converted to uncertainty in flow rate using the calibration curve. The uncertainty in the calibration, normally quoted by the NIST flow calibration facility, is ±0.25% with 99% confidence. Uncertainties in the flow rate produced by the temperature and pressure correction, given by eq (15) result from uncertainties in the temperature and pressure. The uncertainty in pressure is estimated as ±3 mm Hg due to uncertainties in the barometric pressure reading, and uncertainties in measuring the pressure inside the classifier. The uncertainty in temperature is estimated as ±0.5 °C. The resulting uncertainty in volumetric flow rate due to temperature and pressure uncertainties is estimated as ±0.4%. The effect of humidity on the volumetric flow rate is estimated to be 0.2% in section 3.2.2. The sum of all the flow related uncertainties is 1.25% and the sum in quadrature is 0.5%. We use as an overall uncertainty in the volumetric flow rate an intermediate value of ±1.0%.

#### 3.4.3 Uncertainty in Geometric Measurements

The uncertainty in the values of the center rod radius, *r*_1_, the outer cylinder radius, *r*_2_, and the classification length, *L*, listed in table 3 are estimates of how accurately the measurements can be made. For the inner radius, *r*_1_, the uncertainty of ±0.2% represents about ±0.04 mm in diameter, which includes the variability of the diameter over the length of the center rod and the difference between the diameter indicated by the manufacturer (1.874 cm) and the single measurement made during this project (1.870 cm). The uncertainty in *r*_2_ is estimated as 0.3% in a similar manner to *r*_1_, although its larger value represents the increased difficulty in measuring the inner diameter of the cylinder. The uncertainty in length is estimated as 0.5% reflecting both the uncertainty in measuring the length (manufacturer’s measurement was 44.44 cm compared to 44.37 cm measured in this study), as well as the distortion of the electric and flow fields at the entrance and exit of the classifier column.

#### 3.4.4 Uncertainty in Peak Voltage

The uncertainty in voltage corresponding to the peak is estimated as ±0.25% corresponding to ±10 V for the nominal 0.1 μm peak voltage of 3750 V when 333 cm^3^/s sheath air is used. This value of uncertainty was estimated by considering the data used during the repeatability measurement. It represents twice the voltage spread for the second day of the repeatability measurements (the repeatability measurements are shown in table 1). The uncertainty in voltage reading due to calibration accuracy is estimated as ±0.2%. Summing both uncertainty levels, the overall uncertainty in the voltage is estimated as ±0.45%.

#### 3.4.5 Uncertainty in Slip Correction

The uncertainty in the slip correction for 0.1 μm particles is estimated to be ±0.4% based on a recent study by Allen and Raabe [14]. However, there is a 2.4% difference between the slip correction computed by Allen and Raabe [14] for a Knutsen number of 1.3 (0.1 μm diameter sphere at ambient pressure) and their earlier computation [15] based on a reanalysis of Millikan’s oil drop data [18,19]. Allen and Raabe [14] attribute this discrepancy to the difference in the surface accommodation for the solid PSL spheres in their study compared to the Hquid droplet surface in Millikan’s studies. Because of this large difference (2.4%) and because there have been no slip correction measurements on 0.1 μm spheres, an intermediate estimate of the slip correction uncertainty of ±0.9% is used.

#### 3.4.6 Pressure, Temperature, and Viscosity

The effects of the uncertainty in the temperature and pressure measurements on the flow calibration and flow measurements has been included in the flow uncertainty. However, the temperature and pressure also affect the mean free path of the gas eq (18), which in turn affects the slip correction, and the temperature affects the viscosity eq (19). The uncertainty in the viscosity of air itself is about 0.04% [20,21].

#### 3.4.7 Additional Uncertainties—Aerosol Flow Rate and Impurity Effect

All of the uncertainties discussed above directly affect the quantities appearing in the governing equations. The uncertainty associated with varying the aerosol flow rates in tandem is not accounted for by propagating the uncertainty through the governing equations. In fact, as pointed out in sec. 3.2.4, the particle size computed from eqs (1) and (12) is not affected by changing the aerosol flow rates as long as they are kept equal. In section 3.2.4 the uncertainty associated with the aerosol flow rate is estimated to be ±0.5%.

The uncertainty associated with impurities in the water does not affect the measuring accuracy of the classifier itself, but it does lead to a systematic increase in the PSL particle diameter as an aerosol compared to the actual size of the PSL sphere without any impurity coating. The impurity uncertainty is estimated to be 0/–0.3% based on the effect of impurity concentration on the residue thickness together with the effect of an impactor on the PSL particle diameter (sec. 3.3.3).

#### 3.4.8 Total Uncertainty in the Particle Size Measured by the Classifier

All of the various sources of systematic uncertainty in regard to the electrostatic classifier are listed in table 3. A conservative measure of the combined systematic uncertainty is to consider the worst-case situation in which each variable is offset by its uncertainty to produce an extreme value of the diameter. The estimate is made by calculating the diameter using the nominal variable values and comparing to the diameter calculated if all the variables are offset by the magnitude of their uncertainty with the signs chosen so that the total uncertainty is a maximum. The percent change in the diameter is ±2.4%. Adding to this the random error, *R*, an overall error of ±2.5% is obtained.

There is one additional uncertainty that must be included and this is the uncertainty associated with the aerosol flow rate, ±0.5%. Adding this value to the worst case total, we arrive at our best estimate of the uncertamty in measuring particle size with the electrostatic classifier as *U* = ±3.0%.

#### 3.4.9 Total Uncertainty in the Measurement of the 0.1 μm PSL Spheres

To obtain the total uncertainty in sizing the 0.1 μm diameter PSL spheres with the classifier, we must include the impurity effect. While impurities in the water and in the particle suspension do not affect the performance of the classifier, they do cause the size of the PSL sphere to be slightly larger as an aerosol compared to the size of the PSL sphere itself. In this case the error is only in the minus direction; that is, this error causes the measured size to be too large by up to 0.3%. Adding this error to the worst case estimate for *U* given above, we obtain a total uncertainty for the 0.1 μm PSL spheres of +3.0%/−3.3%. This corresponds to the following range in terms of particle diameter:

$$\text{Diameter}=0.1069\begin{array}{l} +0.0032\\ -0.0035 \end{array}\mu m.$$

## 4. Discussion

One way of assessing the validity of the uncertainty estimates is to compare the classifier results for the 0.3 and 1.0 μm SRMs with the certified values. In both cases the diameter obtained by the classifier method is larger than the certified value, by 1.6% for the 0.269 μm SRM and by 1.7% for the 0.895 μm SRM. The important point is that the percent difference between the SRM values and the certified values are smaller than the percent uncertainty (+3.0/−3.3) that we have estimated for the 0.1 μm particle diameter.

In a recent study, Knollenberg [10] summarized other measurements for the same batch of PSL sphere and reported 0.102 μm ± 0.007 μm (Knollenberg, light scattering) and 0.105 μm (Yamada, [11], electron microscopy). There are unresolved issues about the accuracy of size measurements by electron microscopy because of the uncertainties in the determination of the magnification and in defining the edge of the particle [22], Yamada’s study [11] has quantified the effect of the electron beam exposure time on the change in the particle diameter. The good agreement between the classifier measurements and the electron microscopy is encouraging but not conclusive because of the undefined uncertainties in the electron microscopy results.

In KnoUenberg’s study, the light scattering intensity of 0.1 μm PSL sphere is compared with that of 0.269 μm SRM spheres for wavelength large enough that the scattering is in the Rayleigh regime. In this case the primary source of error is the uncertainty in the SRM particle itself. The size reported by Knollenberg [10], 0.102 μm, is outside the uncertainty limits of the classifier measurement; however, the uncertainty limits for the light scattering measurement are broad (±0.007 μm) and include the 0.107 μm average size obtained by the classifier.

To further reduce the uncertainty associated with the classifier method, it is proposed that the classifier be calibrated with the 0.895 μm SRM, which has an uncertainty of ±0.9%. Both the 0.1 μm PSL and the 0.895 μm SRM would be measured using the same flow conditions in the classifier to remove the large flow uncertainty. By analyzing the multiply charged 0.895 μm particles, a high flow rate can be used in the classifier thus minimizing the uncertainties associated with operating the classifier at low flow. It is believed that the uncertainty in the determination of the average particle size for the 0.1 μm PSL can be reduced to about 1.5% by using this procedure.

## Figures and Tables

**Figure 8a:**
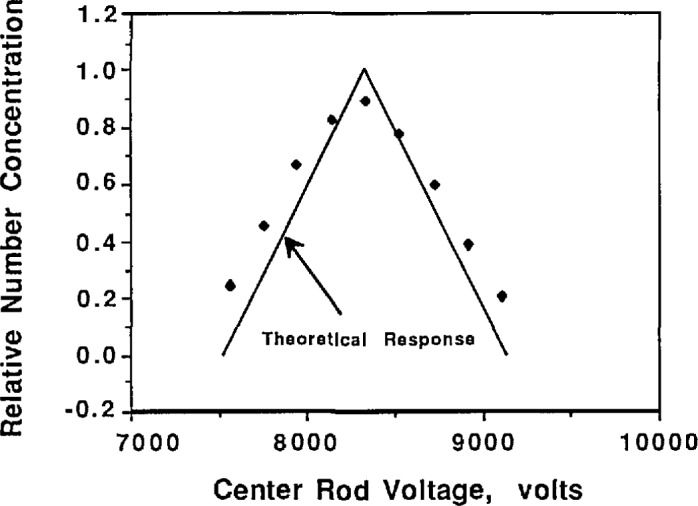
Comparison of experimental and theoretical response of the classifier for 0.269 μm particles using equal aerosol flow rates.

**Figure 8b:**
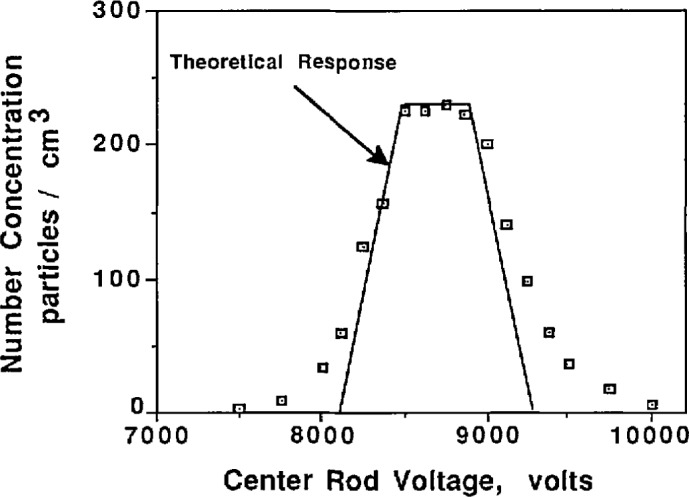
Comparison of experimental and theoretical response of the classifier for 0.269 μm particles using different aerosol flow rates.

**Figure 9a:**
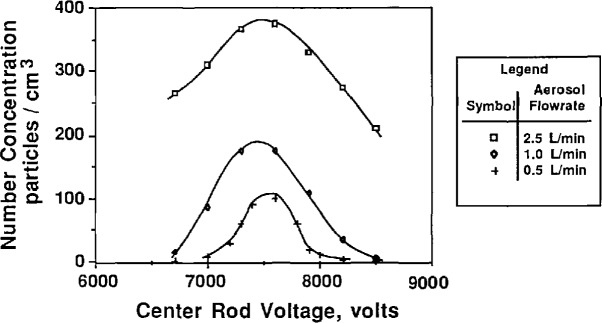
Voltage vs experimental concentration for 0.269 μm particles for three different aerosol flow rates.

**Figure 9b:**
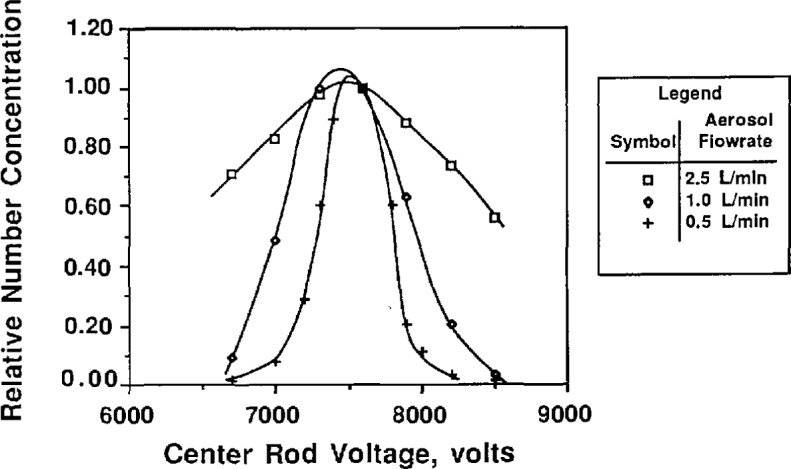
Voltage vs relative concentration for 0.269 μm particles for three different aerosol flow rates.

**Figure 10a:**
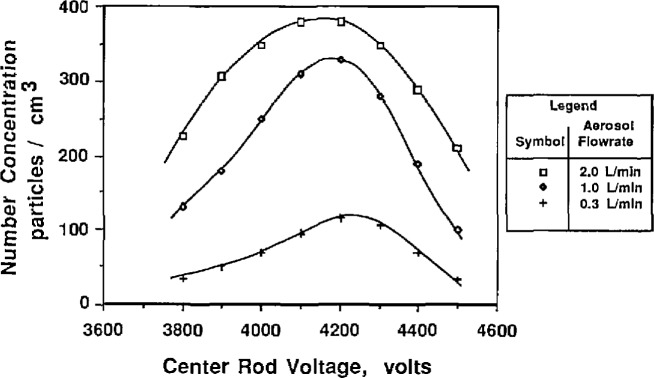
Voltage vs experimental concentration for 0.1 μm particles for three different aerosol flow rates.

**Figure 10b:**
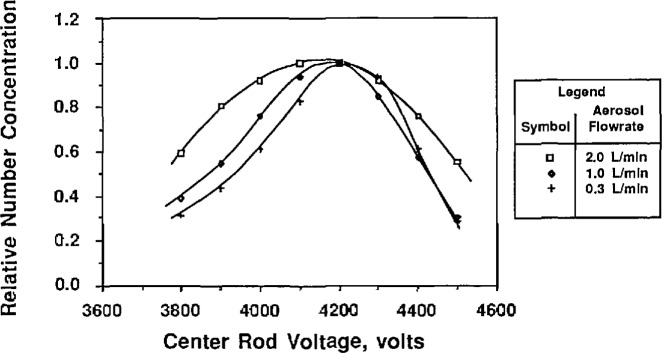
Voltage vs relative concentration for 0.1 μm particles for three different aerosol flow rates.

**Figure 11a:**
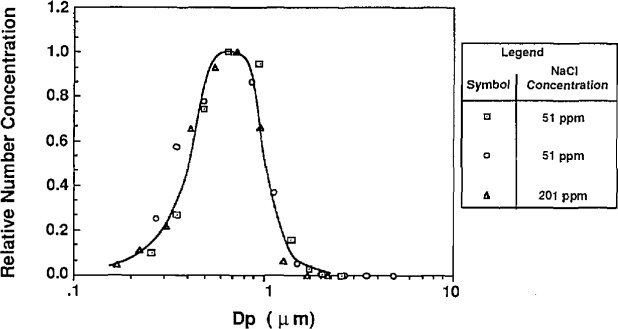
Droplet number distribution with impactor.

**Figure 11b:**
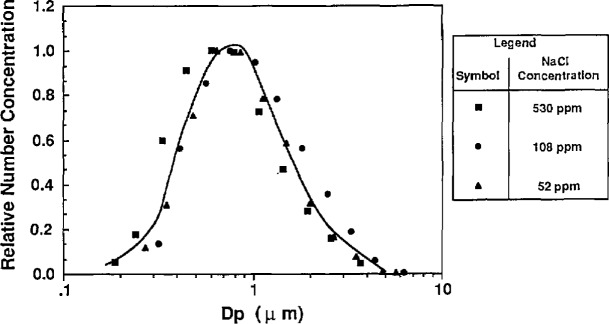
Droplet number distribution without impactor.

**Figure 12a:**
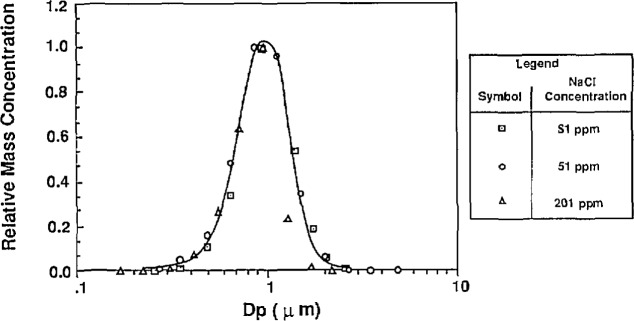
Droplet mass distribution with impactor.

**Figure 12b:**
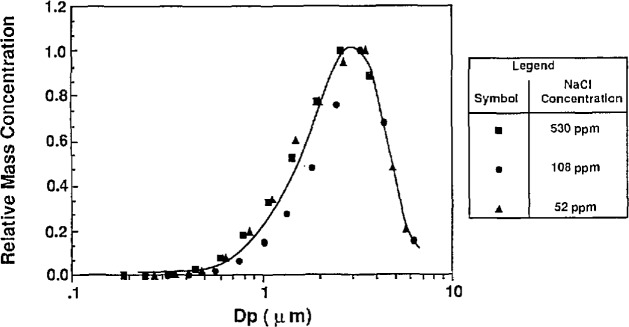
Droplet mass distribution without impactor.

**Figure 13a:**
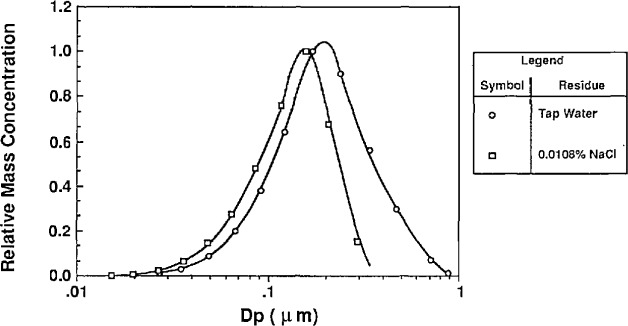
Comparison of residue particles for tap water and 0.010% NaCl.

**Figure 13b:**
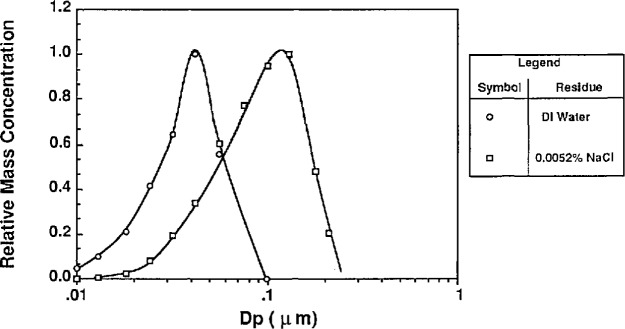
Comparison of residue particles for deionized water and 0.0052% NaCl.

**Table 1:** Repeatability of 0.1 Fim particle diameter measurements

Day 1 Run number	(May 27) Measured diameter μm	Day 2 Run number	(June 1) Measured diameter μm
1	0.1073	1	0.1070
2	0.1072	2	0.1067
3	0.1074	3	0.1069
4	0.1070	4	0.1067
5	0.1069	5	0.1068
6	0.1069	6	0.1068
7	0.1070		
8	0.1068		
$\bar{D}=0.1071\;\text{μm}$		$\bar{D}$ = 0.1068 μm	
*σ_n_*_−1_ = 0.0002 μm (0.2%)		*σ_n_*_−1_ = 0.0001 μm (0.1%)	
Combined Analysis			
$\bar{D}$ = 0.1069 μm			
*σ_n_*_−1_ = 0.0002 μm (0. 2%)			

**Table 2:** Summary of measurements of 0.3 and 0.9 μm Standard Reference Material particles

Standard Reference Material 1691–0.3 μm particles		
Measured diameter		−0.2731 μm
		0.2731
		0.2736
		0.2731
		0.2734
$\bar{D}$	=	0.2733μm
*σ_n_*_−1_	=	+0.0002 μm (0.1%)
*D*_c_^a^	=	0.269 ± 0.007 μm (±2.6%)
$\bar{D}-D_{c}$	=	+0.0043 μm (1.6%)

Standard Reference Material 1690–0.9 μm particles		

Measured diameter		−0.9103 μm
		0.9075
		0.9132
$\bar{D}$	=	0.9103μm
*σ_n_*_−1_	=	0.003 μm (0.3%)
*D*_c_^b^	=	0.895 ± 0.008 μm (±0.9%)
$\bar{D}-D_{c}$	=	+0.015 μm (1.7%)

a Certified diameter for NIST Standard Reference Material 1691.

b Certified diameter for NIST Standard Reference Material 1690.

**Table 3:** Summary of uncertainties^a^ associated with measurement of particle diameter

Variable	Uncertainty in variable	Resulting uncertainty in diameter
*Q*_c_ = sheath air flowrate	1.0%	0.6%
*Q*_m_ = excess air flowrate	1.0%	0.6%
*r*_2_ = outer radius	03%	0.26%
*r*_1_ = inner radius	0.2%	0.16%
*L* = length	0.5%	0.30%
*V* = center rod voltage	0.45%	0.26%
*ϵ* = elementary unit of charge	negligible	0.025%
μ = viscosity of air	0.04%	0.025%
*C* = slip correction	0.9%	0.5%
*T* = temperature	0.2%	0.01%
*P* = pressure	0.4%	0.16%
Worst case estimate from eqs (1) and (12)		±2.4%
Random error, *R*		±0.1%
Residual uncertainty associated with effect of aerosol flowrate on apparent size		±0.5%
Total uncertainty associated with classifier		±3.0%
Impurities related uncertainty		+0%/−0.3%
Total uncertainty—classifier+residue layer		+3.0%/−3.3%

a The uncertainty in particle diameter determined by electrical mobility measurements arises from the uncertainties in the variables used in eqs (1) and (12).

$$Z_{p}=\epsilon C\left(D_{p}\right)/\left(3\pi \mu D_{p}\right)$$ (1)
$$Z_{p}=\left(Q_{c}+Q_{m}\right)\ln\left(r_{2}/r_{1}\right)/\left(4\text{π}VL\right)$$ (12)

## Footnotes

1 Certain commercial equipment, instruments, or materials are identified in this paper to specify adequately the experimental procedure. Such identification does not imply recommendation or endorsement by the National Institute of Standards and Technology, nor does it imply that the materials or equipment identified are necessarily the best available for the purpose.
